# Sarcopenia adversely impacts postoperative complications in living-donor liver transplantation recipients

**DOI:** 10.1038/s41598-021-98399-6

**Published:** 2021-09-28

**Authors:** Mei-Yun Wu, Wei-Xiong Lim, Yu-Fan Cheng, Ching-Di Chang, Hsien-Wen Hsu, Chih-Che Lin, Chao-Long Chen, Wan-Ching Chang, Chun-Yen Yu, Leo Leung-Chit Tsang, Yi-Hsuan Chuang, Hsin-You Ou

**Affiliations:** 1 College of Nursing, Kaohsiung Medical University, Kaohsiung, Taiwan; 2 Department of Nursing, Kaohsiung Chang Gung Memorial Hospital, Chang Gung University College of Medicine, Kaohsiung, Taiwan; 3 Department of Diagnostic Radiology, Kaohsiung Chang Gung Memorial Hospital, Chang Gung University College of Medicine, 123 Ta-Pei Road, Niao-Sung, Kaohsiung, 83305 Taiwan; 4 Department of Surgery, Kaohsiung Chang Gung Memorial Hospital, Chang Gung University College of Medicine, Kaohsiung, Taiwan; 5 Liver Transplantation Program and Departments of Diagnostic Radiology and Surgery, Kaohsiung Chang Gung Memorial Hospital, Chang Gung University College of Medicine, Kaohsiung, Taiwan

**Keywords:** Outcomes research, Liver

## Abstract

Despite technological and immunological innovations, living-donor liver transplant (LDLT) recipients still face substantial risk of postoperative complications. Sarcopenia is being recognized more and more as a biomarker that correlates with poor outcomes in surgical patients. The purpose of this study was to evaluate the relationship between sarcopenia and significant surgical complications in LDLT recipients. This retrospective review included patients who had received LDLT at our institute from 2005 to 2017. Sarcopenia was assessed using the psoas muscle index (PMI) in cross-sectional images. ROC curve analysis was used to determine the ability of PMI to predict postoperative complications. Correlations between major postoperative complications and sarcopenia were evaluated using regression analysis. A total of 271 LDLT recipients were included. No significant differences were found between PMI and major postoperative complications in male patients. Female recipients with major postoperative complications had significantly lower mean PMI values (P = 0.028), and the PMI cut-off value was 2.63 cm^2^/m^2^. Postoperative massive pleural effusion requiring pigtail drainage occurred more frequently in the sarcopenia group than in the non-sarcopenia group (P = 0.003). 1-, 3-, 5- and 10-year overall survival rates in female were significantly poorer in the sarcopenia group (n = 14) compared with the non-sarcopenia group (n = 108), at 92.9% versus 97.2%, 85.7% versus 95.4%, 85.7% versus 92.5% and 70.1 versus 82.0%, respectively (P = 0.041) and 94.6%, 89.9%, 85.9% and 78.5% in male patients. Sarcopenia is associated with a significantly higher risk of major postoperative complications in females. PMI and sarcopenia together are predictive of major postoperative complications and survival rates in female LDLT recipients.

## Introduction

Due to a shortage of deceased donor liver grafts, living donor liver transplantation (LDLT) provides an optimum solution. However, despite substantial technological and immunological innovations, recipients may still experience significant postoperative complications. These complications may include respiratory failure, renal failure, sepsis, abscess formation, ascites and pleural effusion requiring drainage, poor healing/dehiscent wound, gastrointestinal bleeding, bile leakage, internal bleeding, vascular complications, acute rejection, and stroke. Protein-energy malnutrition is common in end-stage liver disease and is closely associated with increased risk of morbidity and mortality after liver transplantation^1,2^.

Sarcopenia, the most common complication in cirrhotic patients, is broadly defined as a significant loss of muscle mass and function. It is recognized as an important independent risk factor for numerous adverse outcomes, including physical disability, falls, osteoporosis, major postoperative complications, prolonged hospital stays, re-admission and death^3–5^. Sarcopenia is also associated with poor outcomes in both non-surgical and surgical patients with serious illnesses^6,7^, and has been shown to be highly predictive of functional impairment, chemotherapy toxicity, and mortality^8,9^. It is also considered one of the main components of cancer cachexia syndrome. Studies have demonstrated that sarcopenia has a negative impact on short-term outcomes after liver resection in patients with liver tumors^10^, and is also associated with mortality in patients with liver cirrhosis^11^.

Multiple previous studies have evaluated sarcopenia for its role in postoperative complications and survival rates after liver transplant^6–9^. However, few studies have evaluated sarcopenia for postoperative complications in LDLT recipients in Asia. Therefore, the present study aimed to investigate the best cutoff value for sarcopenia, focusing on postoperative complications and survival rates in Asian LDLT patients.

## Materials and methods

The protocol for this retrospective study was reviewed and approved by the Research Ethics Committee of Chang Gung Memorial Hospital (IRB no. 201601588B0) and was conducted in accordance with the principles of Declaration of Helsinki and the International Conference on Harmonization for Good Clinical Practice. Informed written consent was obtained from all patients.

### Patients

The data of 271 patients (age > 20 years; 149 males, 122 female) who had undergone liver transplantation at Chang Gung Memorial Hospital (CGMH) between January 2005 and September 2017 were retrieved from the CGMH transplantation database and were included in the analytic sample. Patients without CT cross-sectional images obtained 60 days before transplantation were excluded.

### Computed tomography image analysis

All preoperative CT imaging was obtained using a multi-detector computed tomography scanner (SOMATOM Definition Flash; SIEMENS, Munich, Germany). Standard CT acquisition parameters were as follows:120 kV, variable mA with dose modulation, soft tissue reconstruction algorithm matrix of 512 × 512, field of view (FOV) 30–35 cm, and reconstructed slice thickness 5 mm.

Skeletal muscle areas were analyzed quantitatively at the L3 level by commercial workstation (GE Healthcare Centricity, GE, Chicago, IL, USA). Available CT images were processed through the local Picture Archiving and Communication System (PACS). Manual tracing of the bilateral psoas muscle area (Total psoas muscle area: cm^2^) from the L3 level cross-sectional image was performed by two radiologists. Two observers blinded to patient outcomes read the CT images. The correlation (r) between the total psoas muscle area as measured by two independent observers was 0.97. The total psoas muscle area values were normalized for stature (psoas muscle index PMI: cm^2^/m^2^).

### Morbidity classification

Assessment of post LDLT complications was performed according to the Clavien–Dindo classification. The standardized Clavien–Dindo classification of surgery-related complications is a simple and widely used tool applied to assess and report postoperative complications in general surgery. Prior to any data analysis, we prespecified the primary and secondary outcomes. The primary outcomes were postoperative morbidity occurring within 90 days of transplantation which was scored according to the Clavien–Dindo classification; Complications with a score of > 3a were considered major complications^12^; the secondary outcomes were 1-, 3-, 5- and 10-year overall survival rates.

### Analyzed parameters

Clinical and laboratory data were collected from the CGMH transplantation database. Recipient’s age, body weight, body height, creatinine and albumin levels on admission, Child–Pugh score, model for end-stage liver disease (MELD) scores, graft-to-body-weight-ratio, operative time, cold ischemic time, incidence of postoperative major complications, critical care stay, and overall length of stay were collected. These parameters were compared between the sarcopenic and non-sarcopenic groups. To test the hypothesis that sarcopenic recipients may have greater risk of major postoperative complications, sarcopenia and other variables were compared between recipients with and without major complications. Overall and short-term survival rates were compared between recipients with and without sarcopenia.

### Statistical analysis

Descriptive statistics were calculated for the study cohort. Continuous variables were summarized as mean and standard deviation (mean ± SD). Associations between PMI and other variables were assessed using standard linear regression analysis. Correlations between major postoperative complications and sarcopenia were assessed using binary logistic regression. The impact of sarcopenia on morbidity and mortality was examined using univariable and multivariable logistic regression analyses. Overall and short-term survival rates were analyzed using the non-parametric Kaplan–Meier method. All statistical analyses were 2-tailed, and a value of P < 0.05 was established as statistical significance. All statistical analyses were performed using SAS version 9.4 software (SAS Institute Inc., Cary, NC, USA).

## Results

Of the 271 patients included in the study (Table 1), 45% of the study population was female (n = 122). The mean age at the time of transplant was 51.93 ± 7.4 years. Mean Body Mass Index (BMI) of the cohort was 24.89 ± 4.23, 9.2% were obese and 2.58% were underweight. The mean lab MELD score at the time of transplant was 20.54 ± 10.22. The mean time between CT scan and LDLT is 10.12 ± 7.31 days. The mean PMI was 5.36 ± 2.01 cm^2^/m^2^. A total of 39 patients (14.4%) died during the observation period (6.5 ± 3.57 years). Male patients had a slightly higher mean BMI than females that was still a statistically significant difference (25.08 vs. 24.67; P = 0.014). PMI was significantly higher in males than in females (6.36 ± 1.9 vs. 4.25 ± 1.27; P < 0.0001). Linear regression analysis revealed that PMI correlated significantly (P < 0.05) and negatively with age at transplant and ICU stays; however, it correlated positively with BMI (R-value all < 0.3). Additionally, positive correlations between PMI and albumin, and negative correlations with MELD scores were noted, without statistical significance (Table 1).

**Table 1:** Demographic and clinical characteristics of LDLT recipients.

Characteristic	All patients (n = 271)	Male (n = 149)	Female (n = 122)	P value
Age (mean ± SD)	51.93 ± 7.40	51.61 ± 6.90	52.32 ± 7.98	0.439
BMI	24.89 ± 4.23	25.08 ± 3.55	24.83 ± 4.45	0.602
PMI (cm^2^/m^2^)	5.36 ± 2.01	6.37 ± 1.91	4.29 ± 1.22	< 0.0001
MELD	20.54 ± 10.22	21.79 ± 10.19	19.02 ± 10.10	0.027
Creatinine	0.85 ± 0.39	0.95 ± 0.35	0.73 ± 0.42	0.0001
Albumin	2.92 ± 0.67	2.95 ± 0.73	2.90 ± 0.59	0.511
Total bilirubin	4.57 ± 7.59	4.14 ± 7.19	5.13 ± 8.10	0.291
ICU	20.76 ± 12.35	20.87 ± 13.04	20.65 ± 11.51	0.885
Hospital stay (days)	76.54 ± 41.29	74.87 ± 40.81	78.58 ± 41.95	0.463
**Graft**				0.0001
Left	59	9	50	
Right	212	140	72	
**Etiology of liver disease**				
HBV	58	41	17	
HCV	48	7	41	
HBV/HCV	3	1	2	
Other	53	19	34	
HCC	6	4	2	
T1	3	2	1	1.000
T2	3	2	1	
HBV/HCC	67	60	7	
T1	35	15	20	0.033
T2	32	29	3	
HCV/HCC	29	9	20	
T1	6	2	4	0.270
T2	23	6	17	
HBV/HCV/HCC	7	6	1	
T1	2	2	0	0.495
T2	5	4	1	

*BMI,* Body mass index; *MELD,* Model for end-stage liver disease; *ICU,* Intensive care unit; *HBV,* Hepatitis B virus; *HCV,* Hepatitis B virus; *HCC,* Hepatocellular carcinoa.

During the first 90 days after LDLT, 153 patients presented no or minor complications, whereas 112 patients experienced major complications, including BT, herpes, chest pigtail, TPN, diarrhea, bile leak, pleural effusion and portal vein stenosis. No significant correlations were found between PMI and major complications in male patients (P = 0.709).

**Figure 1:**
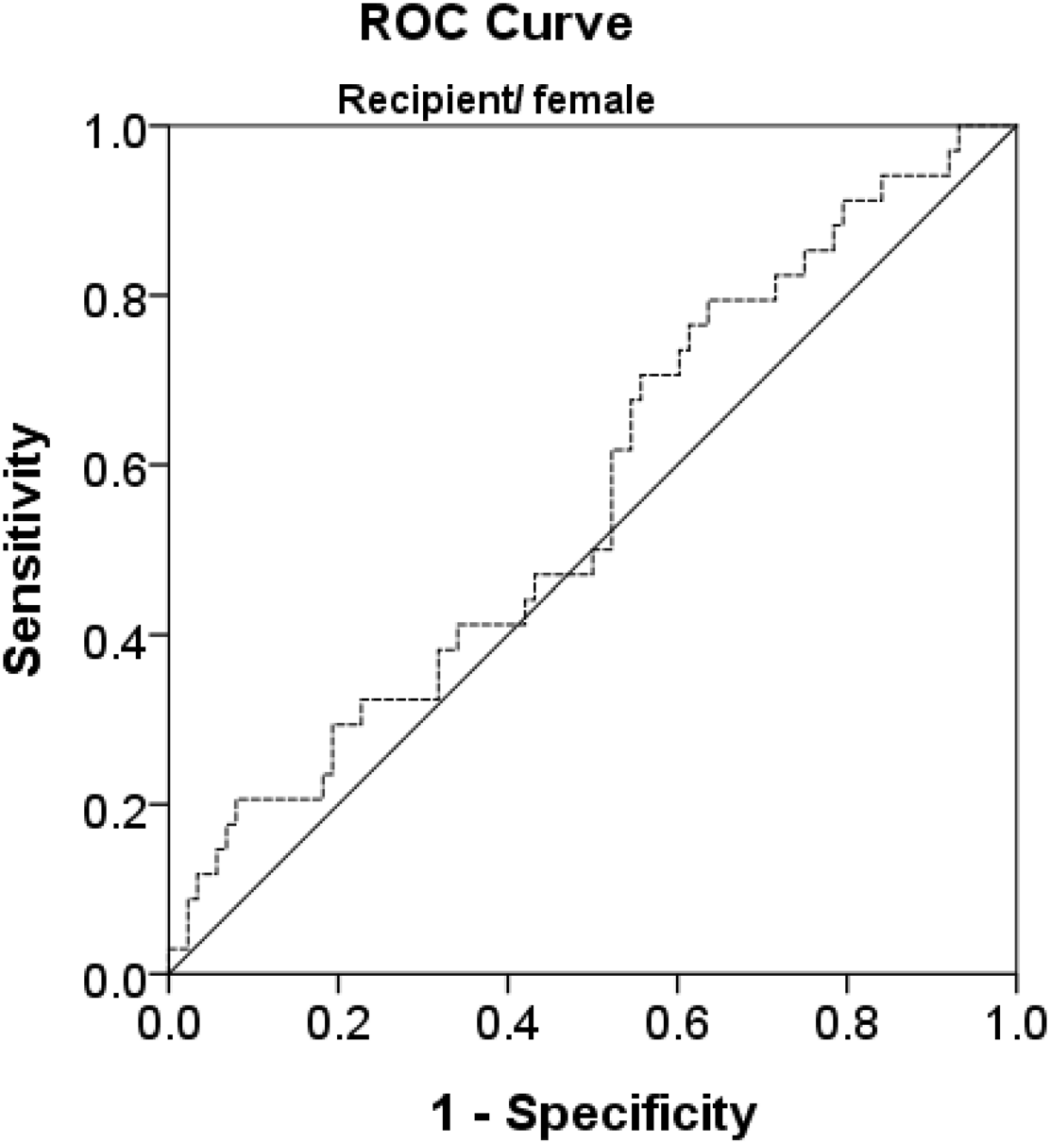
ROC curve analysis revealed PMI correlation with major complications after living donor liver transplant(P = 0.028). The PMI cut-off point as 2.63 (sensitivity = 20.6%, specificity = 92%, AUE = 0.567).

A total of 59 female recipients demonstrated Clavien-Dindo grade ≥ 3 for postoperative complications. The independent samples T-test showed significantly lower mean PMI values (4.04 ± 1.11) in recipient group females with major complications (P = 0.028) than those in the recipient group females without complications (4.52 ± 1.27) (Table 2). ROC curve analysis (Fig. 1) revealed a PMI cut-off point at 2.63 cm^2^/m^2^ (sensitivity = 20.6%, specificity = 92%, AUE = 0.567). Post-operative massive pleural effusion requiring pigtail drainage occurred more frequently in the sarcopenia group than in the non-sarcopenia group (P = 0.003).

**Table 2:** The correlation between postoperative major complications and sarcopenia.

Complication	No/minor complication	Major complication	P value
Male	N = 96	N = 53	
Female	N = 63	N = 59	
Male/PMI	6.324 ± 1.768	6.446 ± 2.155	0.709
Female/PMI	4.521 ± 1.274	4.038 ± 1.108	0.028

*PMI,* Psoas muscle index

**Figure 2:**
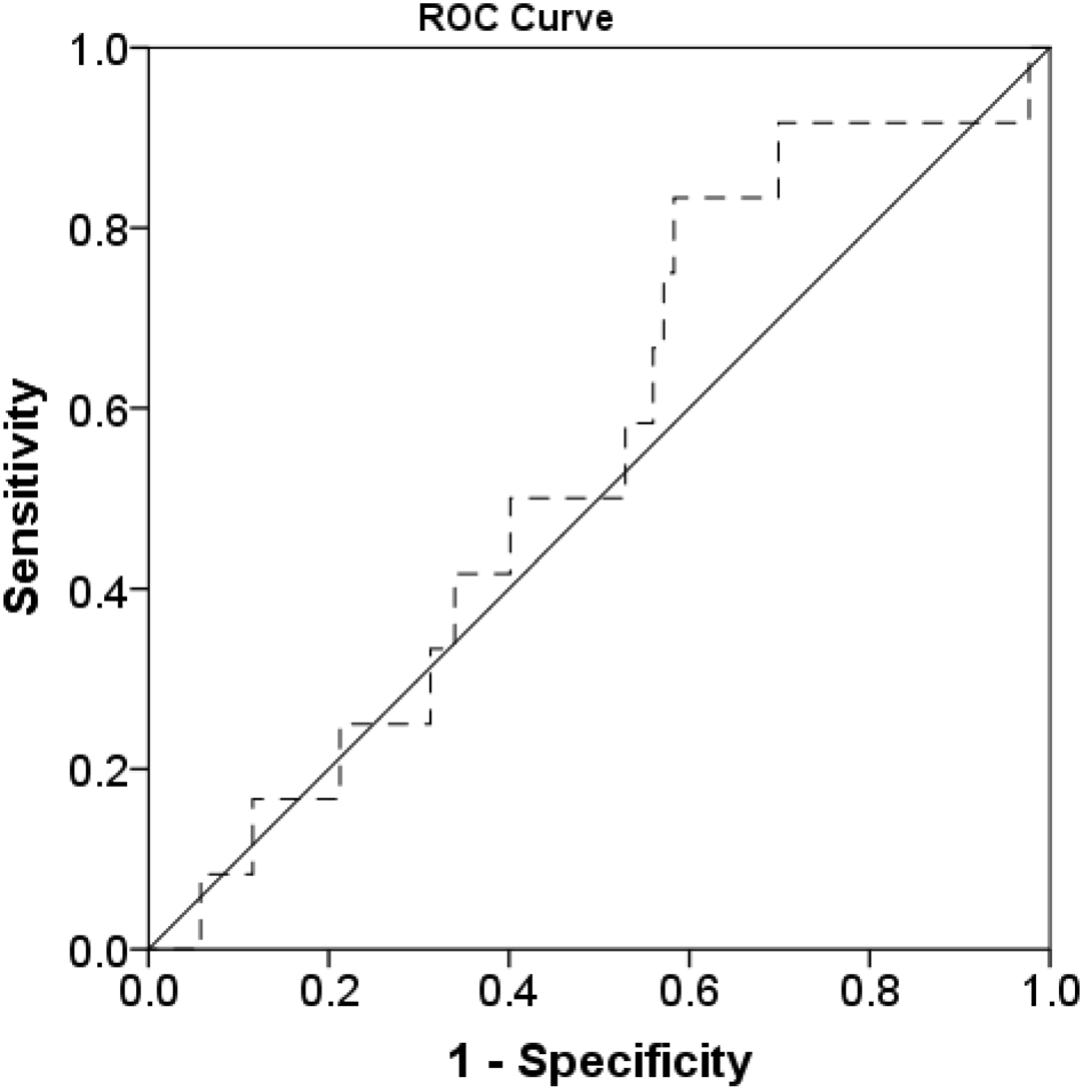
ROC curve analysis revealed PMI correlation with one year mortality after a living donor liver transplant for female PMI and 1-year mortality (sensitivity = 83.3%, specificity = 41.7%, AUE = 0.553).

**Figure 3:**
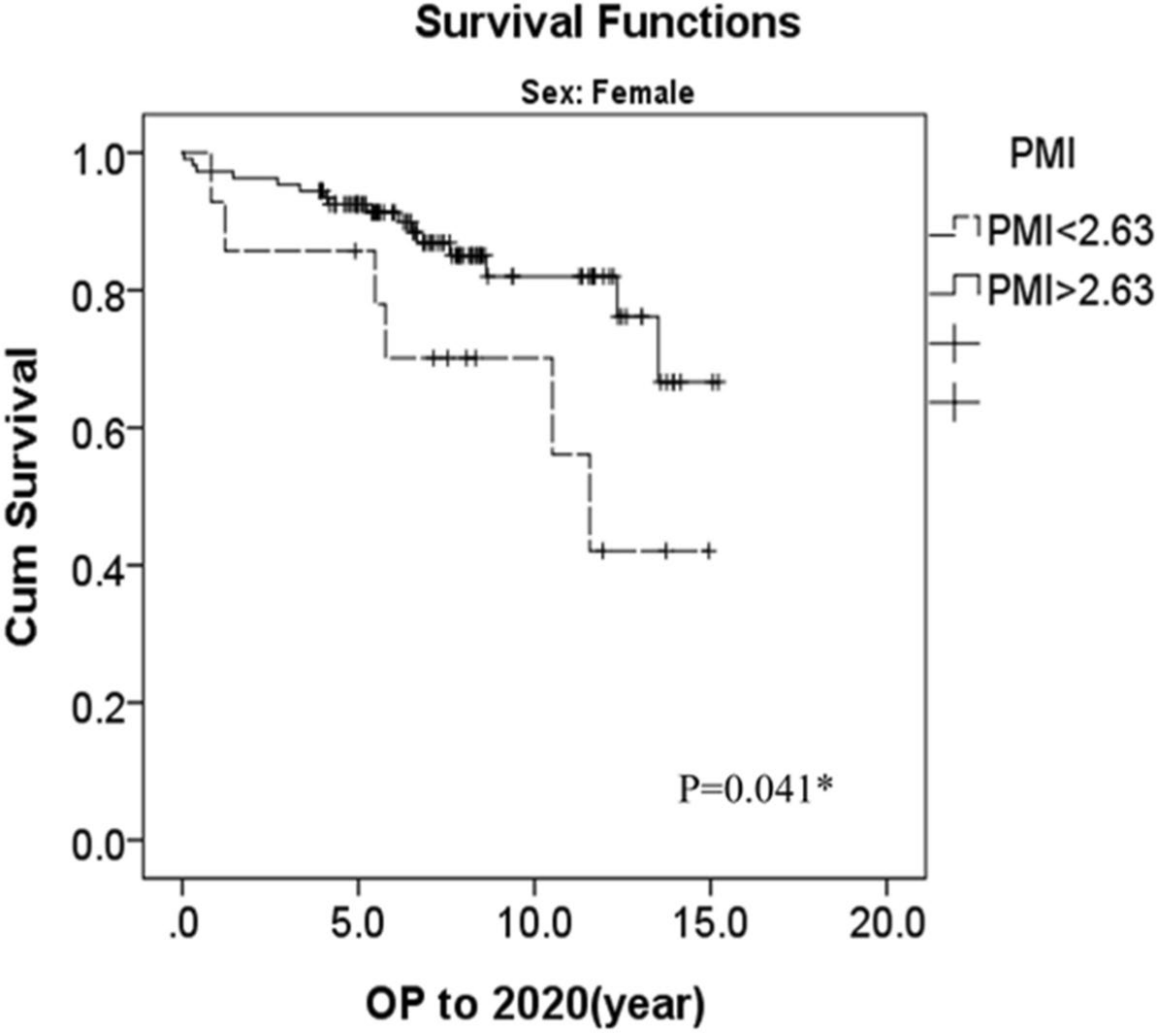
Survival analysis by Kaplan–Meier method.

The 1-, 3-, 5- and 10-year survival rates were 95.5%, 92.6%, 89.7% and 50.1%, respectively. ROC curve analysis revealed PMI correlation with 1 year mortality after a living donor liver transplant for female PMI and 1-year mortality. (sensitivity = 83.3%, specificity = 41.7%, AUE = 0.553) (Fig. 2). Survival analysis using the Kaplan–Meier method (Fig. 3) also showed that 1-, 3-, 5- and 10-years overall survival rates were significantly poorer in the sarcopenia group (n = 14) than in the non-sarcopenia group (n = 108), including 92.9% versus 97.2%, 85.7% versus 95.4%, 85.7% versus 92.5% and 70.1 versus 82.0%, respectively (P = 0.041) (Table 3).

**Table 3:** Comparison of overall survival rate between sarcopenia and non-sarcopenia female patients.

Female/PMI	The number of cases	Mortality	1-year survival (%)	3-year survival (%)	5-year survival (%)	10-year survival (%)
PMI < 2.63	14	6	92.9	85.7	85.7	70.1
PMI > 2.63	108	16	97.2	95.4	92.5	82.0
Overall	122	22				

## Discussion

Results of the present study show that female sarcopenia patients had higher occurrence rates (57%) for major complications after LDLT compared to males and non-sarcopenia patients. This was noted especially in massive pleural effusion (29%), which led to longer ICU stays. These patients all received early drainage with pigtail catheter to improve respiratory function. However, female sarcopenia patients had poor 10-year survival rates (70.1%) compared with those of the non-sarcopenia group (82%). A previous study^13^ reported that pulmonary complications, sepsis, hemorrhage and acute rejection were the major complications observed during the first period of hospitalization. Malnutrition and sarcopenia are common in patients with end-stage liver disease, and the prevalence of sarcopenia in adults before liver transplantation ranged between 14 and 78%. Sarcopenia may lead to higher mortality while patients are on the waiting list for transplant. Recent study results^14^ have shown that sarcopenia is associated with risk of higher morbidity and mortality in cancer patients, postoperative patients, and post-transplant recipients, although the mechanisms are not well understood. Muscle mass functions as a source of amino acids for protein synthesis and gluconeogenesis in stress and starvation^14^. Skeletal muscle loss leads to contractile insufficiency, metabolic impairment, and myokine dysregulation, which contribute to disability, impaired immunity, and risk of sepsis-related death in cirrhotic patients^15^.

Certain measures of muscle mass only account for a single anatomic area, such as single slice imaging or limb anthropometry (CT, MRI). Other measures, including dual X-ray absorptiometry (DXA) and bioelectrical impedance analysis (BIA), are based on total body measures, including fat mass, total body water, total body protein, and bone mineral content. Prakiknjo et al., reported fat free muscle mass in MRI could be as a marker of sarcopenia, which predicts acute-on-chronic liver failure and survival in decompensated cirrhosis^16^ with no radiation exposure and avoids the risk of contrast medium-induced renal failure. However, in the cirrhotic patient, when fluid overload is present, the proportion of total body water is altered, which reduces the accuracy of such methods, leading to overestimation of lean body mass. However, the role of DXA in the evaluation of body composition is well established and remains the leading method for evaluating body composition. In the non-transplant setting, DXA is a more widely available instrument for evaluating muscle quality (total body lean tissue mass or appendicular skeletal muscle mass) non-invasively.

Currently, the most common definition of sarcopenia is appendicular skeletal muscle index more than two SDs below that of typical healthy adults (5.45 kg/m^2^ for females; 7.26 kg/m^2^ for males)^17^. However, recipients included in the present study did not have primary measures of body composition by DXA, therefore we could not report on appendicular skeletal muscle. However, serial CT scan is part of standard clinical care in cirrhotic patients and during liver transplantation assessment, and all recipients in the present study underwent preoperative triphasic CT scan of the abdomen with vascular reconstruction images. These CT images are used to evaluate skeletal muscle mass in adults with liver disease without generating radiation risk or additional cost. Furthermore, MRI and CT are cited as the gold standard for body composition assessment, and they can be used interchangeably to quantify muscle mass^4,18^.

No universal consensus exists on the sarcopenia definition cut-off point based on cross-sectional images. The majority of studies define sarcopenia using the muscle area or skeletal muscle index of the lowest quartile in the patients’ group—below the 5th percentile or < 2SD of the mean in healthy adults/donors groups. The present study used optimum stratification by SAS (version 9.4) to find the most significant P-value using the log-rank χ. statistic to define the sex-specific cut-off points associated with mortality, which was introduced by Prado et al.^19^ in 2008. Optimum stratification solves the threshold value of the continuous covariate that, based on log-rank statistics, best separates patients with sarcopenia from those who did not have sarcopenia for a sufficient time to an event outcome (mortality). These cut-offs were then used to classify patients as those with sarcopenia or those without sarcopenia^19^. Many investigators used the skeletal muscle index cut-off points of Prado et al. (52.4 cm^2^/m^2^ for men and 38.5 cm^2^/m^2^ for women) to define sarcopenia in their studies. Although some studies have cited total skeletal muscle area/index as a more complete measure than psoas muscle area alone, mainly because it is closely related to total body protein and wait-list mortality^20,21^, the psoas muscle area at the L3 lumbar vertebra landmark has also been reported to provide a convenient and reproducible measurement method. A number of studies have demonstrated that the psoas muscle area can accurately estimate the whole-body tissue measurements^22–24^. A recent study by Golse et al.^13^ clarified that the psoas muscle area and psoas muscle index offer better accuracy than the skeletal muscle index in evaluating cirrhotic patients undergoing liver transplantation.

Sarcopenic cancer patients and organ transplant/liver transplant recipients have been shown to have higher morbidity and mortality rates than cancer and transplant recipients without sarcopenia^1,2,6,24,25^. The present study also revealed that sarcopenic LDLT recipients had more postoperative complications with Clavien–Dindo grades ≥ 3. The prevalence of sarcopenia was reported in 17 studies and ranged from 22.2% to nearly 70%. The prevalence greatly depended on the definition used. All studies that reported the prevalence of sarcopenia separately for males and females reported higher prevalence among males. In our study, there is no significant difference between major complications and PMI in male patients, the possible reason may be most of the male patients are a major economic source of the family. Post-operation, they could get full emotional and nutritional support from the whole family. In the female group, some patients with relatively lower education level and sense of illness may cause persisting sarcopenia status after liver transplantation and poor survival rate. Of note, females in the sarcopenia subgroup appeared to have greater vulnerability to major postoperative complications, possibly due to females having lower PMIs than males.

Multiple studies have employed CT to evaluate sarcopenia both in surgical patients using L3/L4 psoas muscle/paravertebral muscles^1^, and in cancer patients using the L3 skeletal muscle index^7–9,19,23,24^. In general, a negative impact associated with sarcopenia has been reported, including increased postoperative complications, increased postoperative infections, decreased survival rates, longer ICU and hospital stays, increased medical costs, poor function status, and increased toxicity from chemotherapy. Pre-liver transplantation sarcopenia and its association with post-transplantation outcomes have been reported. Majority investigator reported sarcopenia associated with poorer survival^2,6^, increase rate of bacteremia and sepsis^26^ and increase post-operative complication^26,27^. However, Montano-Loza et al.^27^ and Aby et al.^28^ reported no differences in survival between sarcopenia and non-sarcopenia patients after liver transplantation. However, Aby et al.^28^ focused on patients with NASH cirrhosis and high MELD scores (72% of recipients were NASH patients). They suggested that the presence of sarcopenia may not impact post-transplant outcomes significantly in patients with high MELD scores who are already significantly decompensated before transplantation. Montano-Loza et al.^27^ stated that the result was possibly due to the exclusion of patients with the most severe sarcopenia during general assessment, or to exclusions for other criteria. In the above two studies, Aby et al. defined sarcopenia by the quartile method, while Montano-Loza et al. defined sarcopenia based on the cutoff values validated in a different population (GI & respiratory tract cancer patients) by optimal stratification statistical method introduced in a previous study^23^.

### Limitations

The present study has several limitations. First, it was a retrospective cohort study using data from only a single center, which narrows generalizability of results. Liver recipients were randomly included within the study period but perioperative management and surgical techniques also have changed during the 11-year study period. Second, measuring muscle mass alone does not account for the loss of muscle function that occurs with sarcopenia. Functional measures such as the walking speed test, 6-min walk test, and grip strength should ideally be done in sarcopenic patients. Fortunately, there is evidence that grip strength correlates significantly with the psoas muscle area^29^. Finally, we did not measure the muscle quality which can be done by measuring intramuscular adipose tissue content. Muscle wasting is characterized by both a reduction in muscle size and an increased proportion of intermuscular and intramuscular fat, and fat infiltration is an additional metabolic abnormality of the depletion process. Sarcopenic obesity, a condition associated with a higher risk of metabolic syndrome and coronary artery disease^30,31^, is known to increase postoperative mobility and mortality.

In conclusion, sarcopenia is associated with major postoperative complications, and patients with postoperative complications have a higher mortality rate. Sarcopenia also has a significant negative impact on transplant patients’ overall survival.
